# *Porphyromonas gingivalis* Induces Increases in Branched-Chain Amino Acid Levels and Exacerbates Liver Injury Through *livh/livk*

**DOI:** 10.3389/fcimb.2022.776996

**Published:** 2022-03-10

**Authors:** Leng Wu, Rui Shi, Huimin Bai, Xingtong Wang, Jian Wei, Chengcheng Liu, Yafei Wu

**Affiliations:** ^1^ State Key Laboratory of Oral Diseases, National Clinical Research Center for Oral Diseases, West China Hospital of Stomatology, Department of Periodontics, Sichuan University, Chengdu, China; ^2^ Department of Stomatology, Tongji Hospital, Tongji Medical College, Huazhong University of Science and Technology, Wuhan, China; ^3^ Department of Periodontics and Oral Mucosal Diseases, The Affiliated Stomatology Hospital of Southwest Medical University, Luzhou, China; ^4^ Department of Hematology, Cancer Center, The First Hospital of Jilin University, Changchun, China

**Keywords:** *Porphyromonas gingivalis*, branched-chain amino acids, non-alcoholic fatty liver disease, dual RNA-sequencing, *livh*, *livk*

## Abstract

*Porphyromonas gingivalis*, a keystone periodontal pathogen, has emerged as a risk factor for systemic chronic diseases, including non-alcoholic fatty liver disease (NAFLD). To clarify the mechanism by which this pathogen induces such diseases, we simultaneously analyzed the transcriptome of intracellular *P. gingivalis* and infected host cells *via* dual RNA sequencing. Pathway analysis was also performed to determine the differentially expressed genes in the infected cells. Further, the infection-induced notable expression of *P. gingivalis livk* and *livh* genes, which participate in branched-chain amino acid (BCAA) transfer, was also analyzed. Furthermore, given that the results of recent studies have associated NAFLD progression with elevated serum BCAA levels, which reportedly, are upregulated by *P. gingivalis*, we hypothesized that this pathogen may induce increases in serum BCAA levels and exacerbate liver injury *via livh/livk*. To verify this hypothesis, we constructed *P. gingivalis livh/livk*-deficient strains (*Δlivk*, *Δlivh*) and established a high-fat diet (HFD)-fed murine model infected with *P. gingivalis*. Thereafter, the kinetic growth and exopolysaccharide (EPS) production rates as well as the invasion efficiency and *in vivo* colonization of the mutant strains were compared with those of the parental strain. The serum BCAA and fasting glucose levels of the mice infected with either the wild-type or mutant strains, as well as their liver function were also further investigated. It was observed that *P. gingivalis* infection enhanced serum BCAA levels and aggravated liver injury in the HFD-fed mice. Additionally, *livh* deletion had no effect on bacterial growth, EPS production, invasion efficiency, and *in vivo* colonization, whereas the *Δlivk* strain showed a slight decrease in invasion efficiency and *in vivo* colonization. More importantly, however, both the *Δlivk* and *Δliv*h strains showed impaired ability to upregulate serum BCAA levels or exacerbate liver injury in HFD-fed mice. Overall, these results suggested that *P. gingivalis* possibly aggravates NAFLD progression in HFD-fed mice by increasing serum BCAA levels, and this effect showed dependency on the bacterial BCAA transport system.

## Introduction

Globally, non-alcoholic fatty liver disease (NAFLD), with a prevalence of approximately 25%, is one of the most common liver diseases (Younossi et al., 2018), and reportedly, it has as one of its pathological features, the excessive accumulation of triglyceride in the liver, owing to metabolic alterations (Zhang et al., 2018a). It has also been observed that this disease can develop into reversible steatosis and non-alcoholic steatohepatitis (NASH), which is a more serious form of NAFLD that shows potential progression to liver cirrhosis or cancer (Friedman et al., 2018). The mechanisms by which NAFLD develops and progresses are extremely complicated. Thus, it has been suggested that several potential risk factors, such as diabetes mellitus and obesity, can lead to its aggravation (Buzzetti et al., 2016; Friedman et al., 2018). Further, increasing evidence from cross-sectional studies, as well as longitudinal and experimental studies, has shown the existence of an association between periodontitis and NAFLD (Weintraub et al., 2019; Chen et al., 2020; Hatasa et al., 2021). Specifically, periodontitis, which is an inflammatory disease that is initiated by the dysbiosis of oral flora, is characterized by the destruction of alveolar bone and connective tissues around the teeth (Liu et al., 2017b; Lu et al., 2020; Hajishengallis and Lamont, 2021). It has also been suggested that *Porphyromonas gingivalis*, a gram-negative anaerobe, is the most important pathogenic bacterium in periodontitis (Lamont et al., 2018; Liu et al., 2021). Furthermore, *P. gingivalis* possesses a plurality of virulence factors that invade periodontal tissues and subsequently enter blood circulation and disseminate into the whole body, increasing the risk of several systemic diseases, notably diabetes, cardiovascular diseases, rheumatoid arthritis, Alzheimer’s disease, and NAFLD (Potempa et al., 2017; Nakahara et al., 2018; Mei et al., 2020; Fitzsimonds et al., 2021). Previous studies have shown that *P. gingivalis* infection aggravates liver inflammation and fibrosis in NASH mouse model induced by high-fat diet (HFD) (Furusho et al., 2013; Nagasaki et al., 2020). Recently, Nagasaki et al. also reported that the elimination of *P. gingivalis*-odontogenic infection inhibits liver inflammation and fibrosis of NASH (Nagasaki et al., 2021). Thus, the mechanisms by which *P. gingivalis* infection leads to liver injury have attracted our attention.

Branched-chain amino acids (BCAAs), which include leucine, isoleucine, and valine, are essential amino acids that mediate the regulation of important hepatic metabolic signaling pathways, such as insulin signaling and glucose regulation (Zhang et al., 2018b). Multiple studies have revealed that elevated circulating BCAA levels are strongly associated with obesity and diabetes, which are the most well-known risk factors for NAFLD (McCormack et al., 2013; Menni et al., 2013; Lynch and Adams, 2014; Nakamura et al., 2014), and in several recent studies, the relationship between BCAAs and NAFLD progression has been demonstrated (Cook et al., 2015; Zhang et al., 2016; Zhao et al., 2020). In particular, the downregulated expression of hepatic BCAA-degrading enzymes has been identified as a hallmark of NAFLD (Mardinoglu et al., 2014; Lake et al., 2015). Moreover, it has also been observed that patients with NASH show higher hepatic BCAA levels than patients with simple steatosis (Lake et al., 2015), and in addition to BCAA-degrading deficiency and fat accumulation in the liver, it has been reported that serum BCAA levels are positively correlated with the severity of NAFLD (Kalhan et al., 2011; Gaggini et al., 2018; Grzych et al., 2020). Recently, Zhao et al. found that BCAAs supplementation in HFD-mice could disrupt hepatic glucose and lipid metabolism and aggravate liver insulin resistance *via* negatively regulating hepatic Akt2 signaling. BCAAs supplementation led to mTORC1-dependent insulin receptor substrate activation, which then block insulin-mediated Akt activation. On the other hand, BCAAs supplementation could inhibit mTORC2 signaling, and subsequently induce Akt2 ubiquitination and degradation *via* promoting the binding of Mul1 and Akt2 (Zhao et al., 2020).

Humans do not synthesize BCAAs, and elevated human serum BCAA levels are strongly associated with gut microbiota (Pedersen et al., 2016). Notably, *Prevotella copri* and *Bacteroides vulgatus*, which have a high potential for BCAA biosynthesis and a low potential for BCAA import, have been identified as the major contributors to increased BCAA levels in humans (Pedersen et al., 2016). Interestingly, a recent study also demonstrated that *P. gingivalis* enhances serum BCAA levels *via* BCAA biosynthesis and consequently, induces insulin resistance in HFD-fed mice (Tian et al., 2020). Furthermore, genetic analysis and transport studies have revealed that the leucine-isoleucine-valine (LIV) system, a member of the ATP-binding cassette (ABC) superfamily of transporters, is required for BCAA transport (Ribardo and Hendrixson, 2011). Therefore, the aim of the present study was to comprehensively analyze the transcriptomes of intracellular *P. gingivalis* and endothelial cells infected by *P. gingivalis* to clarify the mechanism by which this pathogen exacerbates liver injury.

## Materials And Methods

### Bacteria and Cell Culture

*P. gingivalis* ATCC 33277 was cultured anaerobically in trypticase soy broth (TSB) or on TSB-blood agar plates supplemented with 1 g/L yeast extract, 5 μg/mL hemin, and 1 μg/mL menadione at 37°C. When needed, erythromycin (10 μg/mL) was added to the medium to eliminate isogenic mutants. Human umbilical vein endothelial cells (HUVECs) (EA. hy926) were maintained in Dulbecco’s modified Eagle’s medium with fetal bovine serum (10%) (Millipore Sigma, St. Louis, MO, USA) and penicillin/streptomycin (1%) at 37°C in a 5% CO_2_ atmosphere (Wang et al., 2017). The PCR fusion technique and electroporation were utilized to generate the *P. gingivalis* BCAA ABC transporter permease (*livh*)-deficient strain *(Δlivh*) and the ABC-type BCAA transport system periplasmic component (*livk*)-deficient strain *(Δlivk*) *via* homologous recombination, as described in **Figure S1** (Wright et al., 2014). The primers used for mutant construction and confirmation are shown in **Table S1**. The mutants were confirmed *via* PCR and DNA sequencing (**Figure S1**).

HUVECs were seeded in 6-well plates and cultured to 80% confluence. After washing with phosphate-buffered saline (PBS), cells were infected with *P. gingivalis* at a multiplicity of infection of 100 for 2 h at 37°C in 5% CO_2_, unless otherwise stated. Non-adherent bacteria were removed by washing with PBS, and the cell-adherent bacteria were killed using gentamicin (300 μg/mL) and metronidazole (200 μg/mL) for 1 h at 37°C in 5% CO_2_ (Moffatt et al., 2012). For RNA isolation, the cells were washed with PBS and lysed using TRIzol^®^ reagent (Invitrogen, Carlsbad, CA, USA).

### Dual RNA Sequencing

Total RNA was extracted using TRIzol^®^ reagent (Invitrogen, Carlsbad, CA, USA) according to protocols provided by the manufacturer. Further, genomic DNA was removed using the commercially available DNase I system (Qiagen, Germantown, PA, USA), and RNA concentration was measured by determining the A260/A280 ratio using a Nanodrop 2000 system (ThermoFisher, Waltham, MA, USA). RNA integrity was verified *via* 1.5% agarose gel electrophoresis, and qualified RNAs were quantified using a Qubit 3.0 fluorometer with a QubitTM RNA Broad Range Assay Kit (ThermoFisher, Waltham, MA). Furthermore, the RNA samples were analyzed at the Huayin Genome Research Facility (Huayin Health Medical Group Co, Wuhan, China) to determine ribosomal RNA (rRNA) depletion and subsequent RNA sequencing, and rRNA was removed using a Ribo-off rRNA Depletion Kit (Vazyme Biotech Co., Nanjing, China) for humans (Catalog No. N406) and/or bacteria (catalog NO. N407). Additionally, cDNA libraries for Illumina^®^ sequencing were generated using the KC-Digital™ Stranded mRNA Library Prep Kit (Illumina, San Diego, CA, USA). The library products corresponding to 200–250 bps were then enriched, quantified, and sequenced using a Nova-seq 6000 sequencer (Illumina, San Diego, CA, USA) (Westermann et al., 2016; Westermann et al., 2017; Westermann and Vogel, 2018).

Trimmomatic version 0.36 was used to pre-process raw sequencing data to remove low-quality reads and trim adaptor sequences. To eliminate duplication bias introduced during library preparation and sequencing, the clean reads were first clustered according to the unique molecular identifier sequences, the reads in the same cluster were then compared *via* pairwise alignment to generate new sub-clusters based on sequence identity (>95%), and multiple sequence alignments were conducted to acquire one consensus sequence for each sub-cluster. De-duplicated consensus sequences were then mapped to the human or *P. gingivalis* ATCC 33277 genome using STAR 2.5.3a. Feature Counts (Subread-1.5.1; Bioconductor) was used for counting reads mapped to the exon regions of each gene, after which the number of reads per kilobase million was calculated. Further, the edgeR package v3.12.1 was used to identify differentially expressed genes (DEGs) between the infected group and control group. Genes with false discovery rate (FDR) P-values < 0.05 and absolute log_2_FC value ≥ 1 were considered as differentially expressed. Principal-component analysis was conducted *via* the function rda in the Vegan package in R. Kyoto Encyclopedia of Genes and Genomes (KEGG) enrichment analysis was conducted after the DEGs were identified using the KEGG orthology-based annotation system. Thereafter, to statistically test the results of the enrichment analysis, a hypergeometric test was performed; a cutoff P-value of 0.05 was applied for the identification of the enriched KEGG pathways (Nuss et al., 2017; Westermann and Vogel, 2018). Gene Ontology (GO) enrichment analysis for DEGs was carried out by the R/Bioconductor package clusterProfiler, and P < 0.05 was set as the threshold level to determine the significance of the GO terms (Yu et al., 2012).

### Quantitative Reverse Transcription PCR (qRT-PCR)

For the gene expression assay, total mRNA was extracted as mentioned above and reverse transcribed into cDNA using the PrimeScript RT reagent Kit (Takara Bio, Kusatsu, Japan). qRT-PCR was then performed using the SYBR^®^ Premix Ex TaqTM II Kit (Takara Bio, Kusatsu, Japan) or *via* TaqMan Fast Universal Master Mix and TaqMan gene expression assay (ThermoFisher, Waltham, MA, USA) using an Applied Biosystems 7300 system (Foster City, CA, USA) (Liu et al., 2021). PCR amplification was performed as follows: 95°C for 5 min, and 40 cycles at 95°C for 15 s, 56°C for 15 s and 72°C for 15 s, and melting curve analysis. Relative mRNA expression was quantified through the comparative 2^-ΔΔCT^ method with GAPDH as the internal control (Liu et al., 2015). The mRNA expression levels of *livk* and *livh* were measured using 16S rRNA as an internal reference based on the primers listed in **Table S1**.

### Growth Curves of *P. gingivalis*

The overnight culture of *P. gingivalis* was diluted to an optical density at 600 nm (OD_600_) of 0.1 using fresh medium, and then anaerobically cultured at 37°C. The OD_600_ of the bacterial culture was then measured at 2-h intervals for 24 h using a spectrophotometer (Multiskan GO; Thermo Scientific, Inc., Waltham, MA, USA). The experiments were each repeated three times, and growth curves were generated.

### Invasion Assays

Invasion experiments were set up as mentioned in 2.1 except that HUVECs were seeded in 12-well plates (2.0 × 10^5^ cells per well) and cultured to 60% confluence. After antibiotic treatment, the cells were washed with PBS and then lysed with sterile distilled water (1 mL per well).The lysates were diluted and plated on TSB-blood agar. After incubation anaerobically at 37°C for 10 days, the number of bacterial colonies on TSB-Blood agar was counted to determine invasion efficiency, which was expressed as the percentage of the initial inoculum recovered (Moffatt et al., 2012).

### Exopolysaccharide (EPS) Level Detection

The amount of EPS produced by *P. gingivalis* was determined using fluorescent lectins, as described previously (Liu et al., 2017a). Briefly, *P. gingivalis* cells were labeled with Syto-17 (Thermo Fisher, Waltham, MA, USA) and deposited on glass coverslips, while the polysaccharides were labeled with concanavalin A-fluorescein isothiocyanate isomer-I (FITC) and wheat germ agglutinin-FITC (100 μg mL^-1^) for 30 min at room temperature. Thereafter, images were collected *via* laser scanning confocal microscopy (SP8; Leica, Germany) and analyzed using Volocity software version 6.3 (PerkinElmer, Waltham, MA, USA).

### Animal Studies

All the animal procedures applied in the present study were approved by the Ethics Committee of Tongji Hospital, Tongji Medical College, Huazhong University of Science and Technology and the Ethics Committee of West China Hospital of Stomatology, Sichuan University (WCHSIRB-D-2017-071). C57BL/6 mice (male, five-week-old) were housed in specific pathogen-free facilities, and after one week of environmental acclimation, the mice were randomly assigned into five groups of 5 mice each as follows: normal chow (NC) control, HFD, *P. gingivalis* WT infection with HFD (WT + HFD), *P. gingivalis △livh* infection with HFD *(△livh* + HFD), *and P. gingivalis △livk* infection with HFD (*△livk* + HFD). The mice in the HFD group and NC group were administered Research Diet No. D12492and No. D12450J, respectively (**Table S2**). After 12 weeks, the mice were euthanized, and liver and sera samples were harvested. Further, blood samples were drawn *via* cardiac puncture using non-anticoagulant vacuum tubes and 23G1 needles (Tian et al., 2020).

Mice in the *P. gingivalis* infection groups were inoculated with 1 × 10^9^ bacteria in 100 μL PBS with 2% carboxymethyl cellulose (CMC) (Kuboniwa et al., 2017). Infection was repeated once every 3 days for 8 weeks. Conversely, mice in the NC and HFD groups were inoculated with PBS containing 2% CMC as controls. The body weights of the mice were measured at baseline and at the end of the experiment.

### Quantification of *P. gingivalis* in Liver Tissue

Total DNA was isolated from aliquots of liver samples, and the copy number of the *P. gingivalis* genome in the liver DNA samples was determined *via* qPCR using the Wizard^®^ Genomic DNA Purification Kit (Promega, Madison, WI, USA) and the SYBR^®^ Premix Ex TaqTM II Kit (Takara Bio, Kusatsu, Japan). *P. gingivalis-specific* primers are listed in **Table S1**. Amplification reactions were performed using the following cycling parameters: 95°C for 5 min, 40 cycles at 95°C for 10 s, 60°C for 15 s, and 72°C for 15 s. The corresponding copy numbers were calculated using a standard curve (**Figure S2**), generated as previously described (Kuboniwa et al., 2017).

### Glucose, Alanine Transaminase (ALT), Aspartate Aminotransferase (AST), and BCAA Levels in Blood

The mice were fasted for 6 h prior to blood sample collection for the determination of fasting blood glucose (FBG) levels. Specifically, after the fasting, blood samples were collected from the tail vein of the mice and FBG levels were measured using a glucometer (Roche, Basel, Switzerland). Serum was collected after centrifuging the blood samples at 1,000 × *g* for 10 min at 4°C and stored at -80°C until further analysis. Serum AST and ALT levels were also measured using AST and ALT activity assay kits, respectively (Millipore Sigma, St. Louis, MO, USA), while serum BCAA levels were determined using BCAA assay kits (Abcam, Cambridge, MA, USA).

### Statistical Analysis

Statistical analysis was performed using GraphPad Prism software version 7.0 (San Diego, CA, USA). Unpaired two-tailed Student’s t-tests were used to assess differences between groups, while analysis of variance (ANOVA) with Tukey’s test was carried out for multiple comparisons to analyze datasets with more than two groups. Statistical significance was set at P < 0.05. Further, the results were presented as mean ± standard deviation (mean ± SD).

## Results

### Upregulation of Bacterial Genes Related to BCAA Transport Within Endothelial Cells

**Figure 1:**
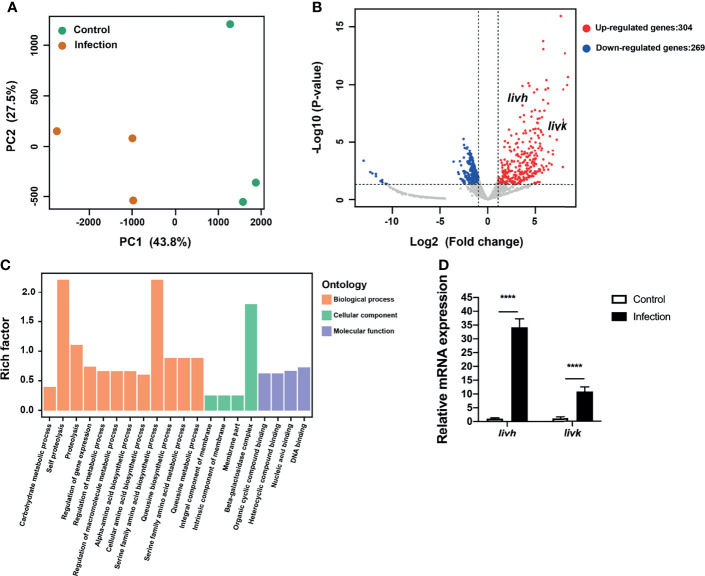
Transcriptional profiling of *P. gingivalis* in HUVECs. **(A)** Transcriptional patterns of intracellular *P. gingivalis* (infection) and *P. gingivalis* culture in the medium (control) showing clear divergence as determined *via* PCA. **(B)** Volcano plot of differentially expressed genes. **(C)** Bar plots of enriched GO terms of upregulated genes in intracellular *P. gingivalis*. **(D)** Detection of the expression levels of *livh* and *livk* in *P. gingivalis*. FC of the expression level of each gene in intracellular *P. gingivalis* (infection) relative to that in the *P. gingivalis* culture medium (control). HUVECs, human umbilical vein endothelial cells; PCA, principal component analysis; FC, fold change. The results presented are the average of three independent experiments and are presented as mean ± SD; ^****^P ≤ 0.0001.

Principal component analysis (PCA) was used to determine the distances between *P. gingivalis* transcriptomes within endothelial cells and the free culture medium. Specifically, the first principal component (PC1), which showed the largest variance (43.8%), separated intracellular *P. gingivalis* and *P. gingivalis* in the culture medium (**Figure 1A**). Additionally, intracellular *P. gingivalis* showed a total of 573 DEGs compared with the control bacteria, and of these DEGs, 304 and 269 were upregulated and downregulated, respectively (**Figure 1B**). The complete list of the DEGs corresponding to intracellular *P. gingivalis* is shown in **Table S3**. GO analysis revealed that upregulated genes were significantly enriched in the GO terms amino acid biosynthetic and metabolic processes (**Figure 1C**). Interestingly, ABC transporter genes, which are critical for the virulence of *P. gingivalis*, were also significantly upregulated in intracellular bacteria (**Table S3**). Further analysis of the expression levels of *PGN_RS06330* (BCAA ABC transporter permease, *liv*h) and *PGN_RS06345* (ABC-type BCAA transport system periplasmic component, *livk*) *via* qRT-PCR confirmed that the expression levels of *livh* and *livk* increased significantly in intracellular *P. gingivalis* (**Figure 1D**). Adaptation through horizontal gene transfer, which is mediated by *tra* gene homologs (Tribble et al., 2007), is regarded as an important process that is required for the survival of *P. gingivalis* within its host. In this regard, it was observed that *P. gingivalis traA*, *traI*, *traG, traN*, *traJ*, *traO*, *traQ*, *traM*, and *traP* genes were significantly upregulated in HUVECs compared with levels in the culture medium (**Table S3**). Further, HcpR, which is necessary for the growth of *P. gingivalis* with nitrite and nitric oxide, was also upregulated (**Table S3**).

### Role of *livh/livk* in Bacterial Growth, Invasion, and EPS Production

**Figure 2:**
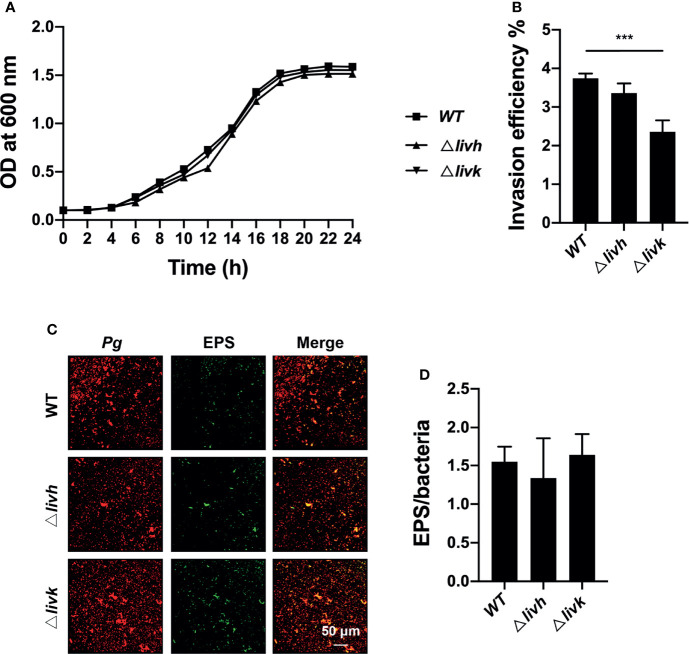
Role of *livh*/*livk* in the growth, invasion efficiency, and EPS production of *P. gingivalis*. **(A)** Growth curve of *P. gingivalis* ATCC 33277 WT, *Δlivh*, and *Δlivk* strain. **(B)** Invasion efficiency of *P. gingivalis Δlivh*/*Δlivk* compared with that of the parental strain. **(C)** Representative CSLM images of EPS (green) labeled with FITC-labeled concanavalin A and wheat germ agglutinin and *P. gingivalis* (red) wild-type, *Δlivh* and *Δlivk*. Scale bar = 50 μm. **(D)** Quantitative determination of EPS using Volocity software version 6.3. CSLM, confocal laser scanning microscopy, EPS, exopolysaccharide, FITC, fluorescein isothiocyanate isomer-I. The results are the average of three independent experiments and are presented as mean ± SD, ^***^P ≤ 0.001.

To explore the role of *livh/livk* in *P. gingivalis*, we further assessed the effect of *livh/livk* deficiency on bacterial growth, invasion, and EPS production *in vitro*. The construction of the growth curve of *P. gingivalis* showed that the growth rates of either the *livh* or *livk* mutant strains were comparable to that of the parental strain (**Figure 2A**). Additionally, the invasion efficiency of the *Δlivk* strain was confirmed using an invasion assay (**Figure 2B**). However, no significant difference in invasion efficiency was observed between the *Δlivh* and WT strains (**Figure 2B**). Moreover, EPS production often plays an important role in biofilm communities developing. Therefore, to assess the role of *livh/livk* in EPS production, *P. gingivalis* was labeled with Syto-17, and EPS was stained with fluorescently labeled lectin concanavalin A and wheat germ agglutinin (**Figure 2C**). Thereafter, the level of EPS was normalized to that of *P. gingivalis*, and it was observed that the rates of production of EPS in *Δlivh* and *Δlivk* were not significantly different from those in the parental strain (**Figure 2D**).

### Eukaryotic Targets of *P. gingivalis* Enriched in the NAFLD Pathway

**Figure 3:**
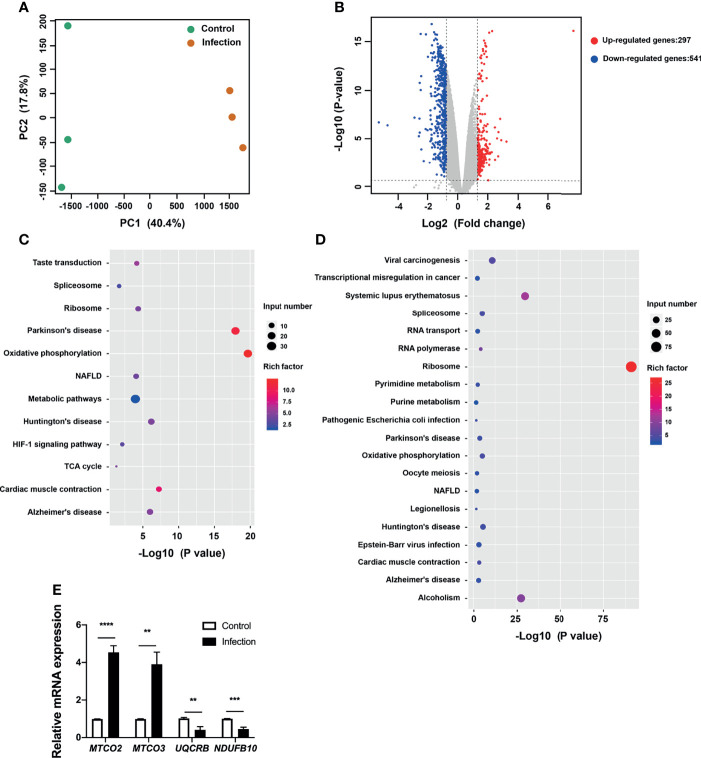
Transcriptional pattern of HUVECs in response to *P. gingivalis* infection. **(A)** Transcriptional pattern of HUVECs infected with *P. gingivalis* (infection) and uninfected cells (control) showing clear divergence as determined *via* PCA. **(B)** Volcano plot of differentially expressed genes in HUVECs. **(C)** KEGG analysis of upregulated genes in HUVECs during infection. **(D)** KEGG analysis of downregulated genes in HUVECs during infection. **(E)** Validation of RNA sequencing data *via* qRT-PCR. The expression of *MTCO2*, *MTCO3*, *UQCRB*, and *NDUFB10* in endothelial cells. FC of the expression of each gene in HUVECs infected with *P. gingivalis* relative to that corresponding to uninfected cells. HUVECs, human umbilical vein endothelial cells; PCA, principal component analysis; FC, fold change; KEGG, Kyoto Encyclopedia of Genes and Genomes. The results are the average of three independent experiments and are presented as mean ± SD. ^**^P ≤ 0.01; ^***^P ≤ 0.001; ^****^P ≤ 0.0001.

To gain insight into the pathways corresponding to the endothelial cells targeted by bacteria, we analyzed the DEGs and enriched KEGG pathways corresponding to HUVECs challenged with *P. gingivalis*. It was observed that the transcriptional landscapes of the infected and uninfected HUVECs showed two independent clusters, indicating distinct gene expression patterns (**Figure 3A**). Additionally, a total of 838 DEGs were detected in *P. gingivalis*-infected HUVECs compared with their uninfected counterparts, and of these, 297 and 541 genes were upregulated and downregulated, respectively (**Figure 3B**). The complete list of DEGs corresponding to the HUVECs is shown in **Table S4**. Further, we annotated the DEGs identified during the comparison of the infected and uninfected HUVECs based on the KEGG pathway. It was observed that both the upregulated and downregulated genes were enriched in pathways associated with chronic systemic diseases, including Parkinson’s disease, Huntington’s disease, NAFLD, and Alzheimer’s disease (**Figures 3C, D**). However, only the upregulated DEGs were found to be enriched in the taste transduction, metabolic, and hypoxia inducible factor 1 (HIF-1) signaling pathways (**Figure 2C**). Moreover, the downregulated DEGs were also enriched in pyrimidine metabolism, purine metabolism, RNA transport, RNA polymerase, viral carcinogenesis, transcriptional misregulation in cancer, systemic lupus erythematosus, and alcoholism pathways (**Figure 3D**). To corroborate the involvement of NAFLD-related genes in HUVECs challenged with *P. gingivalis*, the expression levels of *MTCO2*, *MTCO3*, *UQCRB*, *and NDUFB10* were detected *via* qRT-PCR (**Table 1**). Consistent with the dual RNA sequencing results, *MTCO2* and *MTCO3* were significantly upregulated, whereas *UQCRB* and *NDUFB10* were significantly downregulated in cells infected with *P. gingivalis* (**Figure 3E**).

**Table 1:** Differentially expressed genes of the NAFLD pathway between infected and uninfected human umbilical vein endothelial cells (|log_2_ FC| ≥ 1 and FDR ≤ 0.05).

Gene Symbol	Log_2_ FC	FDR
*MT-CYB*	1.532528324	2.03E-12
*MTCO3P12*	1.294580426	9.23E-11
*MIR6723*	1.362691234	7.13E-06
*MT-CO2*	1.442745846	5.75E-12
*MT-CO1*	1.434791085	2.72E-11
*MT-CO3*	1.230595848	2.39E-10
*MTCO1P2*	1.174011287	0.002827059
*CDC42P6*	-1.784905872	9.93E-06
*COX4I1*	-1.35886734	2.67E-12
*COX6C*	-1.202149761	1.21E-10
*NDUFA8*	-1.083056922	5.67E-08
*UQCRHL*	-1.636421338	2.29E-08
*UQCRB*	-1.186605753	4.31E-10
*NDUFB10*	-1.283988953	7.11E-11
*UQCR10*	-1.215371564	8.66E-07
*NDUFS5*	-1.496650718	8.89E-12
*COX7B*	-1.488988881	2.36E-10

### Bacteria Induces Increases in Circulating BCAA Levels and Liver Injury *via livh/livk*

**Figure 4:**
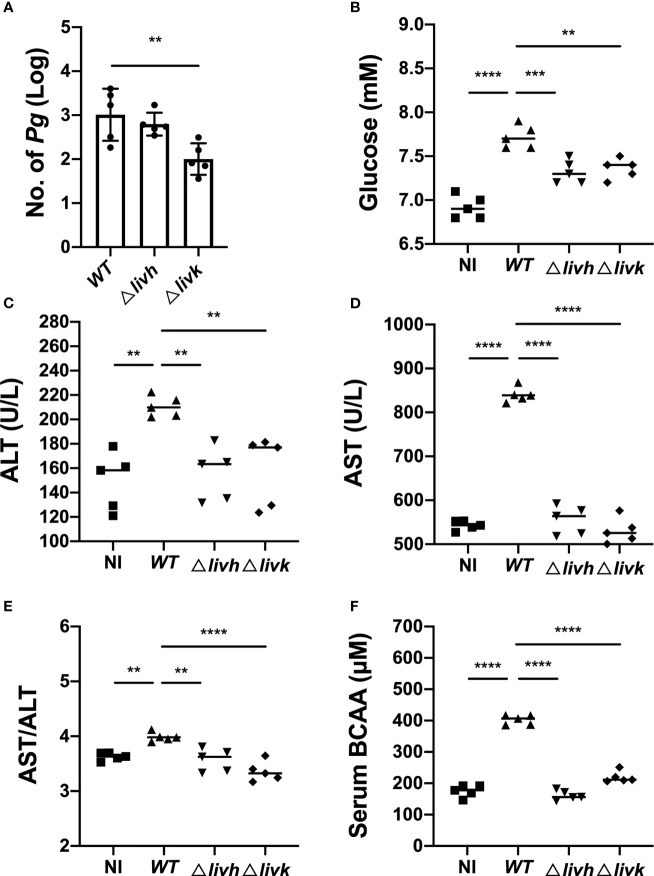
Influence of *P. gingivalis* infection on circulating BCAA levels and liver function. Six-week-old male C57BL/6 mice were infected with 1×10^9^ *P. gingivalis* in 100 μL of PBS containing 2% CMC for 8 weeks. The NC and HFD groups were mock-treated with PBS containing 2% CMC. Four weeks after infection, the mice were euthanized, and liver and serum samples were harvested. **(A)** Colonization of *P. gingivalis* WT, *Δlivh*, and *Δlivk* detected *via* qPCR in the liver tissues of HFD-fed mice. Sera samples from mice were assayed for BCAA and FBG levels, and for the determination of selected liver-specific biochemistries. **(B)** Glucose level, **(C)** ALT level, **(D)** AST level, **(E)** AST/ALT ratio, and **(F)** BCAA level. CMC, carboxymethyl cellulose; BCAA, branched-chain amino acid; NC, normal chow, HFD, high-fat diet. All data represent the mean ± SD for five mice per group. ^**^P ≤ 0.01; ^***^P ≤ 0.001; ^****^P ≤ 0.0001.

To clarify the potential effect of *P. gingivalis* infection on NAFLD, an HFD-fed mouse model was used to confirm the transmission of *P. gingivalis* in the liver, and detect the effect of bacterial infection on circulating BCAA levels, FBG levels, and liver function. As expected, at the end of the experiment, it was observed that the HFD induced an increase in the body weight of the mice, which were unaffected by infection with *P. gingivalis* (**Table S5**). Additionally, the serum levels of FBG, ALT, and AST, as well as the AST/ALT ratios were significantly elevated in mice fed HFD compared with those in mice fed NC (**Figure S3**), suggesting that HFD resulted in impaired liver function in the mice. Consistent with the results of previous studies, *P. gingivalis* was detected in the liver tissue of WT + HFD mice (**Figure 4A**). Further, *P. gingivalis* infection also induced increased serum ALT and AST levels, elevated the AST/ALT ratio, and resulted in higher FBG levels in the HFD-fed mice, confirming that *P. gingivalis* can aggravate HFD-induced liver injury (**Figures 4B** **–E**).

Circulating BCAAs participate in the regulation of liver function, and *livh/livk* has been annotated as a BCAA ABC transporter permease or ABC-type BCAA transport system periplasmic component of *P. gingivalis*. Therefore, we measured serum BCAA levels in mice infected with different strains of *P. gingivalis*. The results showed that *P. gingivalis* infection induced increases in BCAA levels in HFD-fed mice (**Figure 4F**). However, this was compromised by either *Δlivh* or *Δlivk* (**Figure 4F**). Given that the reduced invasion efficiency of the *Δlivk* strain was confirmed *via* an invasion assay (**Figure 2B**), we further investigated the effect of *Δlivh* or *Δlivk* deficiency on *P. gingivalis* liver transmission. We observed that the liver of HFD-fed mice contained less *Δlivk* than the parental strain (**Figure 4A**). Interestingly, FBG, ALT, and AST levels, as well as the AST/ALT ratios in both the *Δlivh* + HFD and *Δlivk* + HFD groups were significantly lower than those in the WT + HFD group. This notwithstanding, the *Δlivh* + HFD and *Δlivk* + HFD groups showed no significant difference (**Figures 4B** **–E**).

## Discussion

This study provides the transcriptional landscape of intracellular *P. gingivalis* as well as that of endothelial cells infected by this bacterium based on dual RNA sequencing. A comparison of the transcriptional profiles of the intracellular bacteria and the free-cultured bacteria cells revealed that *P. gingivalis* infection enhances the expression of BCAA ABC transporter permease (*livh*) and ABC-type BCAA transport system periplasmic component (*livk*) genes to facilitate their survival in host cells. Additionally, both *livh* and *livk* were identified as components of the LIV system, and were found to be responsible for the transport of BCAAs. By analyzing mutants with the deletion of individual genes, we observed that the growth rates of either *Δlivh* or *Δlivk* strains as well as the rate of EPS production in *P. gingivalis*-infected cells were comparable to those in the parental strain. Interestingly, *Δlivk* showed impaired invasion efficiency and *in vivo* colonization. A previous study also showed that *livk*, but not *livh*, is required for commensal colonization by *Campylobacter jejuni* (Ribardo and Hendrixson, 2011). Thus, a possible explanation for this observation is that *livk* may bind to amino acids other than BCAAs, whose transport is required for bacterial survival *in vivo*. To understand different invasion and colonization of *Δlivk* and *Δlivh* mutants, further study such as analyzing the transcriptional profiles of these mutants in HUVEC’s is needed.

Further, transcriptional profiling together with KEGG pathway analysis of HUVECs revealed that *P. gingivalis* induced the differential expression of NAFLD-related genes (e.g., *MTCO2*, *MTCO3*, *UQCRB*, *and NDUFB10*). NAFLD is a condition in which excess fat, not related to alcohol use, is stored in the liver. Further, NAFLD and its progressive inflammatory form, NASH, represent a growing cause of chronic liver injury (Huang et al., 2021), and increasing evidence suggests the existence of a correlation between *P. gingivalis* and NAFLD (Ding et al., 2019). Yoneda et al. first observed that the detection rate of *P. gingivalis* in the saliva is significantly higher in NAFLD patients than that in healthy controls (46.7% vs. 21.7%, odds ratio: 3.16) (Yoneda et al., 2012). Furusho et al. also reported the detection of *P. gingivalis* in 21 out of 40 liver biopsy specimens from NASH patients. They also reported that *P. gingivalis*-positive cases showed significantly higher fibrosis scores than *P. gingivalis*-negative cases (Furusho et al., 2013). Consistent with these previous findings, Nakahara et al. recently identified a significant correlation between NAFLD progression and the titer of serum antibodies against *P. gingivalis* (Nakahara et al., 2018).

Dual RNA-seq has the potential to provide novel insights into the host–pathogen interaction *via* simultaneously determining the transcriptomes of host and pathogen. However, dual RNA-seq is complex experiments. Factors such as heterogeneity in cell populations and infection, differences in transcriptome stabilization, RNA extraction, library preparation, and sequencing may induce batch effects. To obtain accurate representative genomes of the host and pathogen, high sequencing depth is required. Besides, dual RNA-seq crucially depends on proper statistical analysis to determine DEGs during infection. Currently, three popular analysis, EdgeR, DESeq2 and limma/voom, perform well. DESeq generally appears to be more conservative and edgeR more liberal in its p-value calculations. In this study, the unique molecular identifier sequences were introduced to optimize the de-duplication, and DEGs was identified using the edgeR package. In addition to the validation of the sequencing results through qRT-PCR for *in vitro* infection, *Δlivk* and *Δlivh* mutants was constructed and *in vivo* experiments were performed to demonstrate our hypothesis.

Animal studies have also shown that *P. gingivalis* accelerates NAFLD progression in HFD-fed mice. Specifically, Kuraji et al. showed that experimental periodontitis induced by *P. gingivalis* leads to the progression of NASH. It has also been reported that increased levels of endotoxins resulting from *P. gingivalis* infection possibly contribute to NASH progression (Kuraji et al., 2016). Nakahara et al. also observed that fibrosis and steatosis are more severe in HFD-fed mice infected with *P. gingivalis* than in mice without *P. gingivalis* infection, and their further metabolome analysis suggested that the alteration in fatty acid metabolism seems to play a considerable role in the fibrosis progression of NAFLD (Nakahara et al., 2018). Moreover, in HFD-fed mice, intravenous injection of sonicated *P. gingivalis* aggravates NAFLD and changes the expression levels of genes associated with lipid and glucose metabolism in the liver (Sasaki et al., 2018). Mechanistically, *P. gingivalis odontogenic* infection possibly aggravates NASH *via* the upregulation of the lipopolysaccharide and toll-like receptor pathway and free fatty acid-induced NOD-, LRR-, and pyrin domain-containing protein 3 inflammasome activation (Furusho et al., 2013). In this study, we identified LIV as a novel mechanism by which *P. gingivalis* induces increases in BCAA levels and exacerbates liver injury in HFD-fed mice. This finding is consistent with an earlier study, which showed that elevated circulating BCAA levels are significantly related to metabolic diseases, such as insulin resistance and NAFLD (Sunny et al., 2015). It has also been reported that periodontal infection with *P. gingivalis* results in increased circulating BCAA levels and aggravates insulin resistance in mice fed HFD (Tian et al., 2020). Moreover, BCAA aminotransferase deficiency reduces *P. gingivalis-induced* serum BCAA levels and insulin resistance in mice fed HFD (Tian et al., 2020). However, the data from this study should be interpreted with some caution. The repeated oral administration of *P. gingivalis* has also been shown to result in the alteration of gut microbiota, which reportedly, is associated with elevated serum BCAA levels (Saad et al., 2016; Yoon, 2016; Sato et al., 2017). Possibly, mice can also ingest plenty *P. gingivalis* that can affect gut microbiota. Therefore, we could not rule out the possibility that the oral administration of *P. gingivalis* possibly led to changes in gut microbiota characteristics, thus indirectly affecting serum BCAA levels. Moreover, whether *P. gingivalis* accelerates liver injury by enhancing the levels of circulating BCAAs still needs to be further confirmed *in vivo*.

Although this study as well as others showed that *P. gingivalis* induced upregulation of BCAA serum levels in mice, the underlying mechanism is still unclear. Reportedly, BCAA biosynthesis and transportation are key factors in the modulation of bacteria-related intracellular and extracellular BCAA concentrations (Cai et al., 2020; Wendisch, 2020). The overexpression of BCAA-related synthetic pathway genes and BCAA importer genes as well as the deletion of BCAA exporter genes can result in the accumulation of bacterial intracellular BCAAs (Chen et al., 2015; Zhu et al., 2018; Cai et al., 2020). For instance, it has been observed that *Bacillus licheniformis* DW2 YhdG acts as an exporter of BCAAs, and the deletion of the *ydhG* gene improves intracellular BCAA accumulation, while overexpression of the importer genes, *yvbW* and *braB*, increases and decreases intracellular and extracellular BCAA concentrations, respectively (Cai et al., 2020). According to KEGG database, *P. gingivalis* possesses a biosynthetic pathway and transport system (LivKHGMF) of BCAA. *PGN_RS05180* encodes branched-chain amino acid aminotransferase (BCAT), a key enzyme of BCAA synthesis. Multiple sequence alignments show that *P. gingivalis* BCAT contains the conserved domains and active catalytic site of bacterial branched-chain amino acid transaminase. Tian et al. found that *Δbcat* strain loses this ability to increase plasma level of BCAAs. Moreover, *P. copri* and *B. vulgatus*, the major contributors to increased BCAA levels in humans, have a high potential for BCAA biosynthesis. The amino acid sequences of BCAT in *P. gingivalis*, *P. copri* and *B. vulgatus* are highly homologous. These findings suggest that *P. gingivalis* has a strong BCAA synthesis. Intriguingly, the results of this study showed an obvious upregulation of two components of the BCAA transportation system (*livk* and *livh*) of intercellular *P. gingivalis*, as well as different expression levels for the genes associated with the NAFLD pathway in HUVECs infected with *P. gingivalis*. More importantly, we observed that both *livh* and *livk* deletion reduced the ability of *P. gingivalis* to induce increases in serum BCAA, ALT, and AST levels in mice fed HFD. Thus, we speculated that the LIV system of *P. gingivalis* may aggravate NAFLD and NASH by inducing increases in circulating BCAA levels. It is likely that *P. gingivalis* elevate BCAA serum levels through biosynthesizing BCAAs, and then exporting the biosynthesized BCAAs to extracellular environments such as blood circulation. However, it is still unclear whether the effects of *P. gingivalis* on BCAA levels and liver injury are related to specific components of the LIV system. Therefore, the effects of LIV, especially *livh* and *livk*, on BCAA levels and NALFD signaling pathways still require further investigation. For instance, comparing the intracellular and extracellular BCAA concentrations in *P. gingivalis* mutants (*Δbcat*, *Δlivk* and *Δlivh*) under various stresses is needed to validate the role of BCAT and LIV system of *P. gingivalis* in modulating its intracellular and extracellular BCAA concentrations, as well as to explore the regulators of BCAA synthesis and transportation.

## Data Availability Statement

The original contributions presented in the study are included in the article/**Supplementary Material**. The RNA sequencing data are deposited in Gene Expression Omnibus (GEO) database under the accession number GSE184085 (https://www.ncbi.nlm.nih.gov/geo/query/acc.cgi?acc=GSE184085). Further inquiries can be directed to the corresponding author.

## Ethics Statement

All the animal procedures applied in the present study were approved by the Ethics Committee of Tongji Hospital, Tongji Medical College, Huazhong University of Science and Technology and the Ethics Committee of West China Hospital of Stomatology, Sichuan University (WCHSIRB-D-2017-071). And this has been stated in Line 378-382.

## Author Contributions

LW and RS contributed to the study conception and design, data acquisition, analysis, and interpretation, and in the drafting the of the manuscript. HB, XW, and JW contributed to data acquisition, analysis, and interpretation. CL and YW contributed to the study conception and design, data acquisition, analysis, and interpretation, and the drafting and critical revision of the manuscript. All the authors approved the final version of the manuscript and agreed to be accountable for all aspects of the work.

## Funding

This study was supported by the National Natural Science Foundation of China (grant number 82170970 to YW; grant number 81600871 to CL).

## Conflict of Interest

The authors declare that the research was conducted in the absence of any commercial or financial relationships that could be construed as a potential conflict of interest.

## Publisher’s Note

All claims expressed in this article are solely those of the authors and do not necessarily represent those of their affiliated organizations, or those of the publisher, the editors and the reviewers. Any product that may be evaluated in this article, or claim that may be made by its manufacturer, is not guaranteed or endorsed by the publisher.

## Supplementary Material

Supplementary Figure 1 Construction and confirmation of *P. gingivalis Δlivh* and *Δlivk* strain.

Supplementary Figure 2 qPCR standard curve of *P. gingivalis.*

Supplementary Figure 3 Effect of HFD on liver function. Serum **(A)** glucose, **(B)** ALT, **(C)** AST levels, and **(D)** AST/ALT ratio in HFD-fed mice were compared with those in NC-fed mice. HFD, high-fat diet; NC, normal chow. **P ≤ 0.01; ***P ≤ 0.001; ****P ≤ 0.0001.

Supplementary Table 2 The compositions of D12492 and D12450J.

Supplementary Table 3 Differentially expressed genes of intracellular *P. gingivalis* compared with *P. gingivalis* cultured in medium.

Supplementary Table 4 Differentially expressed genes of human umbilical vein endothelial cells (HUVECs) infected with *P. gingivalis* compared with uninfected HUVECs.

## Abbreviations

NAFLD, non-alcoholic fatty liver disease; NASH, non-alcoholic steatohepatitis; HFD, high-fat diet; BCAAs, branched-chain amino acids; EPS, exopolysaccharide; LIV, leucine-isoleucine-valine; ABC, adenosine 5′-triphosphate binding cassette; TSB, trypticase soy broth; HUVEC, human umbilical vein endothelial cells; PBS, phosphate buffered solution; DEGs, differentially expressed genes; FDR, false discovery rate; FC, fold-change; KEGG, Kyoto Encyclopedia of Genes and Genomes; GO, Gene Ontology; FITC, fluorescein isothiocyanate isomer-I; NC, normal chew; CMC, carboxymethyl cellulose; ALT, alanine transaminase; AST, aspartate aminotransferase; ANOVA, analysis of variance; SD, standard deviation. PCA, principal component analysis; PC1, first principal component; HIF-1, hypoxia inducible factor 1; qRT-PCR, quantitative reverse transcription PCR; qPCR, quantitative polymerase chain reaction; CSLM, confocal laser scanning microscopy; FBG, fasting blood glucose.

## References

[B1] Buzzetti E. Pinzani M. Tsochatzis E. A. (2016). The Multiple-Hit Pathogenesis of Non-Alcoholic Fatty Liver Disease (NAFLD). Metabolism 65, 1038–1048. doi: 10.1016/j.metabol.2015.12.012 26823198

[B2] Cai D. Zhu J. Li Y. Li L. Zhang M. Wang Z.. (2020). Systematic Engineering of Branch Chain Amino Acid Supply Modules for the Enhanced Production of Bacitracin From Bacillus Licheniformis. Metab. Eng. Commun. 11, e00136. doi: 10.1016/j.mec.2020.e00136 32637317 PMC7326738

[B3] Chen C. Li Y. Hu J. Dong X. Wang X. (2015). Metabolic Engineering of Corynebacterium Glutamicum ATCC13869 for L-Valine Production. Metab. Eng. 29, 66–75. doi: 10.1016/j.ymben.2015.03.004 25769288

[B4] Chen Y. Yang Y. C. Zhu B. L. Wu C. C. Lin R. F. Zhang X. (2020). Association Between Periodontal Disease, Tooth Loss and Liver Diseases Risk. J. Clin. Periodontol 47, 1053–1063. doi: 10.1111/jcpe.13341 32621350

[B5] Cook J. R. Langlet F. Kido Y. Accili D. (2015). Pathogenesis of Selective Insulin Resistance in Isolated Hepatocytes. J. Biol. Chem. 290, 13972–13980. doi: 10.1074/jbc.M115.638197 25873396 PMC4447970

[B6] Ding L. Y. Liang L. Z. Zhao Y. X. Yang Y. N. Liu F. Ding Q. R.. (2019). Porphyromonas Gingivalis-Derived Lipopolysaccharide Causes Excessive Hepatic Lipid Accumulation *via* Activating NF-kappaB and JNK Signaling Pathways. Oral. Dis. 25, 1789–1797. doi: 10.1111/odi.13153 31283861

[B7] Fitzsimonds Z. R. Liu C. Stocke K. S. Yakoumatos L. Shumway B. Miller D. P.. (2021). Regulation of Olfactomedin 4 by *Porphyromonas Gingivalis* in a Community Context. ISME J. 15, 2627–2642. doi: 10.1038/s41396-021-00956-4 33731837 PMC8397782

[B8] Friedman S. L. Neuschwander-Tetri B. A. Rinella M. Sanyal A. J. (2018). Mechanisms of NAFLD Development and Therapeutic Strategies. Nat. Med. 24, 908–922. doi: 10.1038/s41591-018-0104-9 29967350 PMC6553468

[B9] Furusho H. Miyauchi M. Hyogo H. Inubushi T. Ao M. Ouhara K.. (2013). Dental Infection of Porphyromonas Gingivalis Exacerbates High Fat Diet-Induced Steatohepatitis in Mice. J. Gastroenterol. 48, 1259–1270. doi: 10.1007/s00535-012-0738-1 23307045

[B10] Gaggini M. Carli F. Rosso C. Buzzigoli E. Marietti M. Della Latta V.. (2018). Altered Amino Acid Concentrations in NAFLD: Impact of Obesity and Insulin Resistance. Hepatology 67, 145–158. doi: 10.1002/hep.29465 28802074

[B11] Grzych G. Vonghia L. Bout M. A. Weyler J. Verrijken A. Dirinck E.. (2020). Plasma BCAA Changes in Patients With NAFLD Are Sex Dependent. J. Clin. Endocrinol. Metab. 105, dgaa175. doi: 10.1210/clinem/dgaa175 32271385

[B12] Hajishengallis G. Lamont R. J. (2021). Polymicrobial Communities in Periodontal Disease: Their Quasi-Organismal Nature and Dialogue With the Host. Periodontol 2000 86, 210–230. doi: 10.1111/prd.12371 33690950 PMC8957750

[B13] Hatasa M. Yoshida S. Takahashi H. Tanaka K. Kubotsu Y. Ohsugi Y.. (2021). Relationship Between NAFLD and Periodontal Disease From the View of Clinical and Basic Research, and Immunological Response. Int. J. Mol. Sci. 22 (7), 3728. doi: 10.3390/ijms22073728 PMC8038294 33918456

[B14] Huang D. Q. El-Serag H. B. Loomba R. (2021). Global Epidemiology of NAFLD-Related HCC: Trends, Predictions, Risk Factors and Prevention. Nat. Rev. Gastroenterol. Hepatol. 18, 223–238. doi: 10.1038/s41575-020-00381-6 33349658 PMC8016738

[B15] Kalhan S. C. Guo L. Edmison J. Dasarathy S. McCullough A. J. Hanson R. W.. (2011). Plasma Metabolomic Profile in Nonalcoholic Fatty Liver Disease. Metabolism 60, 404–413. doi: 10.1016/j.metabol.2010.03.006 20423748 PMC2950914

[B16] Kuboniwa M. Houser J. R. Hendrickson E. L. Wang Q. Alghamdi S. A. Sakanaka A.. (2017). Metabolic Crosstalk Regulates Porphyromonas Gingivalis Colonization and Virulence During Oral Polymicrobial Infection. Nat. Microbiol. 2, 1493–1499. doi: 10.1038/s41564-017-0021-6 28924191 PMC5678995

[B17] Kuraji R. Ito H. Fujita M. Ishiguro H. Hashimoto S. Numabe Y. (2016). Porphyromonas Gingivalis Induced Periodontitis Exacerbates Progression of Non-Alcoholic Steatohepatitis in Rats. Clin. Exp. Dent. Res. 2, 216–225. doi: 10.1002/cre2.41 29744170 PMC5839206

[B18] Lake A. D. Novak P. Shipkova P. Aranibar N. Robertson D. G. Reily M. D.. (2015). Branched Chain Amino Acid Metabolism Profiles in Progressive Human Nonalcoholic Fatty Liver Disease. Amino Acids 47, 603–615. doi: 10.1007/s00726-014-1894-9 25534430 PMC4329055

[B19] Lamont R. J. Koo H. Hajishengallis G. (2018). The Oral Microbiota: Dynamic Communities and Host Interactions. Nat. Rev. Microbiol. 16, 745–759. doi: 10.1038/s41579-018-0089-x 30301974 PMC6278837

[B20] Liu C. Miller D. P. Wang Y. Merchant M. Lamont R. J. (2017a). Structure-Function Aspects of the Porphyromonas Gingivalis Tyrosine Kinase Ptk1. Mol. Oral. Microbiol. 32, 314–323. doi: 10.1111/omi.12173 27498608 PMC5293697

[B21] Liu C. Mo L. Niu Y. Li X. Zhou X. Xu X. (2017b). The Role of Reactive Oxygen Species and Autophagy in Periodontitis and Their Potential Linkage. Front. Physiol. 8, 439. doi: 10.3389/fphys.2017.00439 28690552 PMC5481360

[B22] Liu C. Niu Y. Zhou X. Xu X. Yang Y. Zhang Y.. (2015). Cell Cycle Control, DNA Damage Repair, and Apoptosis-Related Pathways Control Pre-Ameloblasts Differentiation During Tooth Development. BMC Genomics 16, 592. doi: 10.1186/s12864-015-1783-y 26265206 PMC4534026

[B23] Liu C. Stocke K. Fitzsimonds Z. R. Yakoumatos L. Miller D. P. Lamont R. J. (2021). A Bacterial Tyrosine Phosphatase Modulates Cell Proliferation Through Targeting RGCC. PLoS Pathog. 17, e1009598. doi: 10.1371/journal.ppat.1009598 34015051 PMC8172045

[B24] Lu L. Yakoumatos L. Ren J. Duan X. Zhou H. Gu Z.. (2020). JAK3 Restrains Inflammatory Responses and Protects Against Periodontal Disease Through Wnt3a Signaling. FASEB J. 34, 9120–9140. doi: 10.1096/fj.201902697RR 32433819 PMC7501269

[B25] Lynch C. J. Adams S. H. (2014). Branched-Chain Amino Acids in Metabolic Signalling and Insulin Resistance. Nat. Rev. Endocrinol. 10, 723–736. doi: 10.1096/fj.201902697RR 25287287 PMC4424797

[B26] Mardinoglu A. Agren R. Kampf C. Asplund A. Uhlen M. Nielsen J. (2014). Genome-Scale Metabolic Modelling of Hepatocytes Reveals Serine Deficiency in Patients With Non-Alcoholic Fatty Liver Disease. Nat. Commun. 5, 3083. doi: 10.1038/ncomms4083 24419221

[B27] McCormack S. E. Shaham O. McCarthy M. A. Deik A. A. Wang T. J. Gerszten R. E.. (2013). Circulating Branched-Chain Amino Acid Concentrations Are Associated With Obesity and Future Insulin Resistance in Children and Adolescents. Pediatr. Obes. 8, 52–61. doi: 10.1111/j.2047-6310.2012.00087.x 22961720 PMC3519972

[B28] Mei F. Xie M. Huang X. Long Y. Lu X. Wang X.. (2020). Porphyromonas Gingivalis and Its Systemic Impact: Current Status. Pathogens 9, 944. doi: 10.3390/pathogens9110944 PMC7696708 33202751

[B29] Menni C. Fauman E. Erte I. Perry J. R. Kastenmuller G. Shin S. Y.. (2013). Biomarkers for Type 2 Diabetes and Impaired Fasting Glucose Using a Nontargeted Metabolomics Approach. Diabetes 62, 4270–4276. doi: 10.2337/db13-0570 23884885 PMC3837024

[B30] Moffatt C. E. Inaba H. Hirano T. Lamont R. J. (2012). Porphyromonas Gingivalis SerB-Mediated Dephosphorylation of Host Cell Cofilin Modulates Invasion Efficiency. Cell Microbiol. 14, 577–588. doi: 10.1111/j.1462-5822.2011.01743.x 22212282 PMC3449298

[B31] Nagasaki A. Sakamoto S. Arai T. Kato M. Ishida E. Furusho H.. (2021). Elimination of Porphyromonas Gingivalis Inhibits Liver Fibrosis and Inflammation in NASH. J. Clin. Periodontol. 48 (10), 1367–1378. doi: 10.1111/jcpe.13523 34250613

[B32] Nagasaki A. Sakamoto S. Chea C. Ishida E. Furusho H. Fujii M.. (2020). Odontogenic Infection by Porphyromonas Gingivalis Exacerbates Fibrosis in NASH *via* Hepatic Stellate Cell Activation. Sci. Rep. 10, 4134. doi: 10.1038/s41598-020-60904-8 32139740 PMC7058079

[B33] Nakahara T. Hyogo H. Ono A. Nagaoki Y. Kawaoka T. Miki D.. (2018). Involvement of Porphyromonas Gingivalis in the Progression of Non-Alcoholic Fatty Liver Disease. J. Gastroenterol. 53, 269–280. doi: 10.1007/s00535-017-1368-4 28741270

[B34] Nakamura H. Jinzu H. Nagao K. Noguchi Y. Shimba N. Miyano H.. (2014). Plasma Amino Acid Profiles Are Associated With Insulin, C-Peptide and Adiponectin Levels in Type 2 Diabetic Patients. Nutr. Diabetes 4, e133. doi: 10.1038/nutd.2014.32 25177913 PMC4183973

[B35] Nuss A. M. Beckstette M. Pimenova M. Schmuhl C. Opitz W. Pisano F.. (2017). Tissue Dual RNA-Seq Allows Fast Discovery of Infection-Specific Functions and Riboregulators Shaping Host-Pathogen Transcriptomes. Proc. Natl. Acad. Sci. U. S. A. 114, E791–E800. doi: 10.1073/pnas.1613405114 28096329 PMC5293080

[B36] Pedersen H. K. Gudmundsdottir V. Nielsen H. B. Hyotylainen T. Nielsen T. Jensen B. A.. (2016). Human Gut Microbes Impact Host Serum Metabolome and Insulin Sensitivity. Nature 535, 376–381. doi: 10.1038/nature18646 27409811

[B37] Potempa J. Mydel P. Koziel J. (2017). The Case for Periodontitis in the Pathogenesis of Rheumatoid Arthritis. Nat. Rev. Rheumatol 13, 606–620. doi: 10.1038/nrrheum.2017.132 28835673

[B38] Ribardo D. A. Hendrixson D. R. (2011). Analysis of the LIV System of Campylobacter Jejuni Reveals Alternative Roles for LivJ and LivK in Commensalism Beyond Branched-Chain Amino Acid Transport. J. Bacteriol 193, 6233–6243. doi: 10.1128/JB.05473-11 21949065 PMC3209225

[B39] Saad M. J. Santos A. Prada P. O. (2016). Linking Gut Microbiota and Inflammation to Obesity and Insulin Resistance. Physiol. (Bethesda) 31, 283–293. doi: 10.1152/physiol.00041.2015 27252163

[B40] Sasaki N. Katagiri S. Komazaki R. Watanabe K. Maekawa S. Shiba T.. (2018). Endotoxemia by Porphyromonas Gingivalis Injection Aggravates Non-Alcoholic Fatty Liver Disease, Disrupts Glucose/Lipid Metabolism, and Alters Gut Microbiota in Mice. Front. Microbiol. 9, 2470. doi: 10.3389/fmicb.2018.02470 30405551 PMC6207869

[B41] Sato K. Takahashi N. Kato T. Matsuda Y. Yokoji M. Yamada M.. (2017). Aggravation of Collagen-Induced Arthritis by Orally Administered *Porphyromonas Gingivalis* Through Modulation of the Gut Microbiota and Gut Immune System. Sci. Rep. 7, 6955. doi: 10.1038/s41598-017-07196-7 28761156 PMC5537233

[B42] Sunny N. E. Kalavalapalli S. Bril F. Garrett T. J. Nautiyal M. Mathew J. T.. (2015). Cross-Talk Between Branched-Chain Amino Acids and Hepatic Mitochondria Is Compromised in Nonalcoholic Fatty Liver Disease. Am. J. Physiol. Endocrinol. Metab. 309, E311–E319. doi: 10.1152/ajpendo.00161.2015 26058864 PMC4537921

[B43] Tian J. Liu C. Zheng X. Jia X. Peng X. Yang R.. (2020). *Porphyromonas Gingivalis* Induces Insulin Resistance by Increasing BCAA Levels in Mice. J. Dent. Res. 99, 839–846. doi: 10.1177/0022034520911037 32176550

[B44] Tribble G. D. Lamont G. J. Progulske-Fox A. Lamont R. J. (2007). Conjugal Transfer of Chromosomal DNA Contributes to Genetic Variation in the Oral Pathogen *Porphyromonas Gingivalis* . J. Bacteriol 189, 6382–6388. doi: 10.1128/JB.00460-07 17573478 PMC1951918

[B45] Wang H. X. Gao X. W. Ren B. Cai Y. Li W. J. Yang Y. L.. (2017). Comparative Analysis of Different Feeder Layers With 3T3 Fibroblasts for Culturing Rabbits Limbal Stem Cells. Int. J. Ophthalmol. 10, 1021–1027. doi: 10.18240/ijo.2017.07.01 28730101 PMC5514260

[B46] Weintraub J. A. Lopez Mitnik G. Dye B. A. (2019). Oral Diseases Associated With Nonalcoholic Fatty Liver Disease in the United States. J. Dent. Res. 98, 1219–1226. doi: 10.1177/0022034519866442 31369716 PMC6755718

[B47] Wendisch V. F. (2020). Metabolic Engineering Advances and Prospects for Amino Acid Production. Metab. Eng. 58, 17–34. doi: 10.1016/j.ymben.2019.03.008 30940506

[B48] Westermann A. J. Barquist L. Vogel J. (2017). Resolving Host-Pathogen Interactions by Dual RNA-Seq. PLoS Pathog. 13, e1006033. doi: 10.1371/journal.ppat.1006033 28207848 PMC5313147

[B49] Westermann A. J. Forstner K. U. Amman F. Barquist L. Chao Y. Schulte L. N.. (2016). Dual RNA-Seq Unveils Noncoding RNA Functions in Host-Pathogen Interactions. Nature 529, 496–501. doi: 10.1038/nature16547 26789254

[B50] Westermann A. J. Vogel J. (2018). Host-Pathogen Transcriptomics by Dual RNA-Seq. Methods Mol. Biol. 1737, 59–75. doi: 10.1007/978-1-4939-7634-84 29484587

[B51] Wright C. J. Xue P. Hirano T. Liu C. Whitmore S. E. Hackett M.. (2014). Characterization of a Bacterial Tyrosine Kinase in Porphyromonas Gingivalis Involved in Polymicrobial Synergy. Microbiologyopen 3, 383–394. doi: 10.1002/mbo3.177 24811194 PMC4082711

[B52] Yoneda M. Naka S. Nakano K. Wada K. Endo H. Mawatari H.. (2012). Involvement of a Periodontal Pathogen, Porphyromonas Gingivalis on the Pathogenesis of Non-Alcoholic Fatty Liver Disease. BMC Gastroenterol. 12, 16. doi: 10.1186/1471-230X-12-16 22340817 PMC3305584

[B53] Yoon M. S. (2016). The Emerging Role of Branched-Chain Amino Acids in Insulin Resistance and Metabolism. Nutrients 8 (7), 405. doi: 10.3390/nu8070405 PMC4963881 27376324

[B54] Younossi Z. Anstee Q. M. Marietti M. Hardy T. Henry L. Eslam M.. (2018). Global Burden of NAFLD and NASH: Trends, Predictions, Risk Factors and Prevention. Nat. Rev. Gastroenterol. Hepatol. 15, 11–20. doi: 10.1038/nrgastro.2017.109 28930295

[B55] Yu G. Wang L. G. Han Y. He Q. Y. (2012). Clusterprofiler: An R Package for Comparing Biological Themes Among Gene Clusters. OMICS 16, 284–287. doi: 10.1089/omi.2011.0118 22455463 PMC3339379

[B56] Zhang F. Hu Z. Li G. Huo S. Ma F. Cui A.. (2018a). Hepatic CREBZF Couples Insulin to Lipogenesis by Inhibiting Insig Activity and Contributes to Hepatic Steatosis in Diet-Induced Insulin-Resistant Mice. Hepatology 68, 1361–1375. doi: 10.1002/hep.29926 29637572

[B57] Zhang Z. Y. Monleon D. Verhamme P. Staessen J. A. (2018b). Branched-Chain Amino Acids as Critical Switches in Health and Disease. Hypertension 72, 1012–1022. doi: 10.1161/HYPERTENSIONAHA.118.10919 30354823

[B58] Zhang F. Zhao S. Yan W. Xia Y. Chen X. Wang W.. (2016). Branched Chain Amino Acids Cause Liver Injury in Obese/Diabetic Mice by Promoting Adipocyte Lipolysis and Inhibiting Hepatic Autophagy. EbioMedicine 13, 157–167. doi: 10.1016/j.ebiom.2016.10.013 27843095 PMC5264279

[B59] Zhao H. Zhang F. Sun D. Wang X. Zhang X. Zhang J.. (2020). Branched-Chain Amino Acids Exacerbate Obesity-Related Hepatic Glucose and Lipid Metabolic Disorders *via* Attenuating Akt2 Signaling. Diabetes 69, 1164–1177. doi: 10.2337/db19-0920 32184272

[B60] Zhu J. Cai D. Xu H. Liu Z. Zhang B. Wu F.. (2018). Enhancement of Precursor Amino Acid Supplies for Improving Bacitracin Production by Activation of Branched Chain Amino Acid Transporter BrnQ and Deletion of Its Regulator Gene Lrp in *Bacillus Licheniformis* . Synth Syst. Biotechnol. 3, 236–243. doi: 10.1016/j.synbio.2018.10.009 30417137 PMC6215969

