# Supplementary figures and images for: Porphyromonas gingivalis Induces Increases in Branched-Chain Amino Acid Levels and Exacerbates Liver Injury Through livh/livk
Source: Front Cell Infect Microbiol. 2022 Mar 10;12:776996. doi: 10.3389/fcimb.2022.776996 (PMC8961321; doi:10.3389/fcimb.2022.776996)

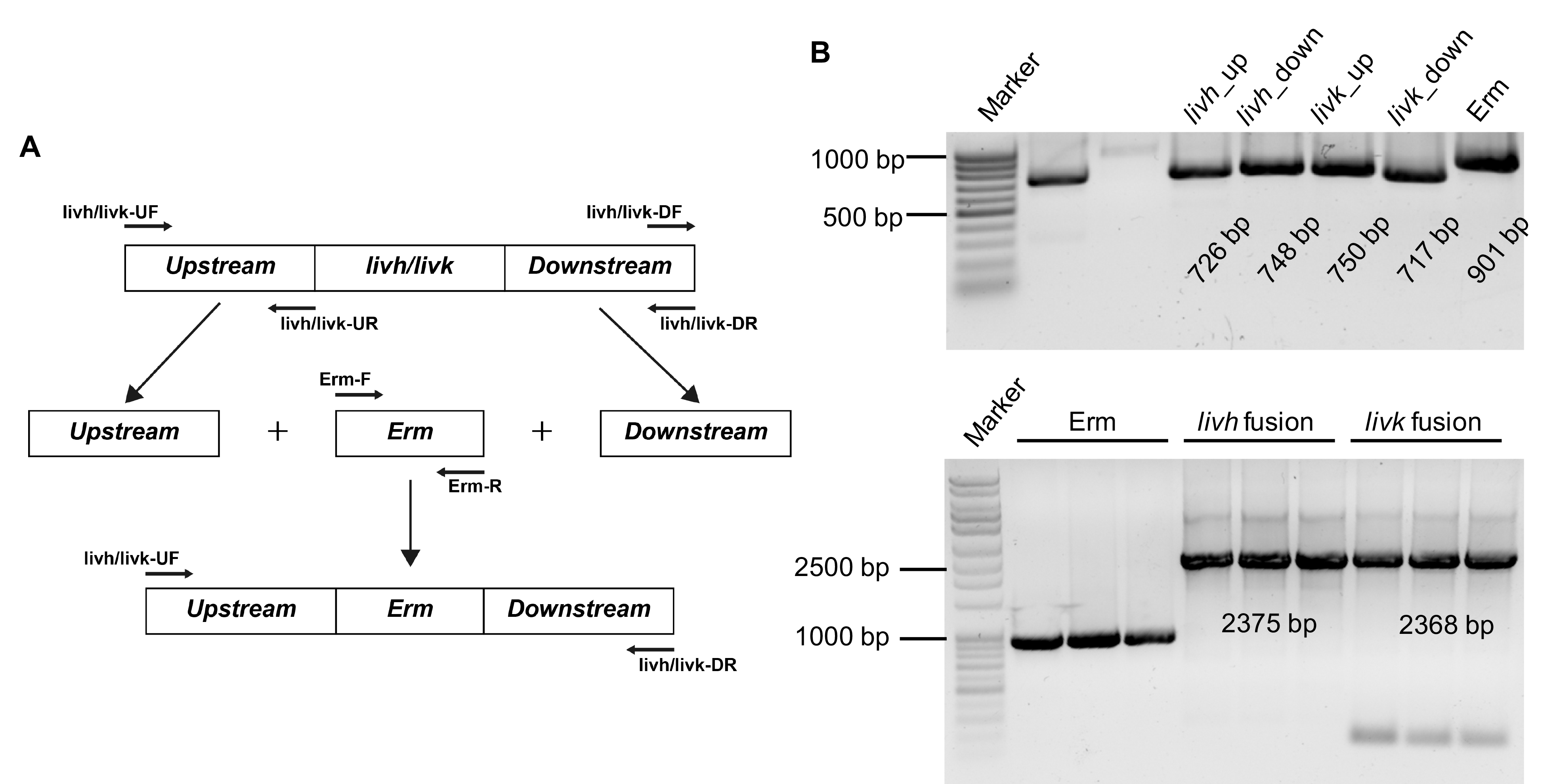

Supplement: Supplementary Figure 1 — Construction and confirmation of P. gingivalis Δlivh and Δlivk strain. [file Image_1.tif]

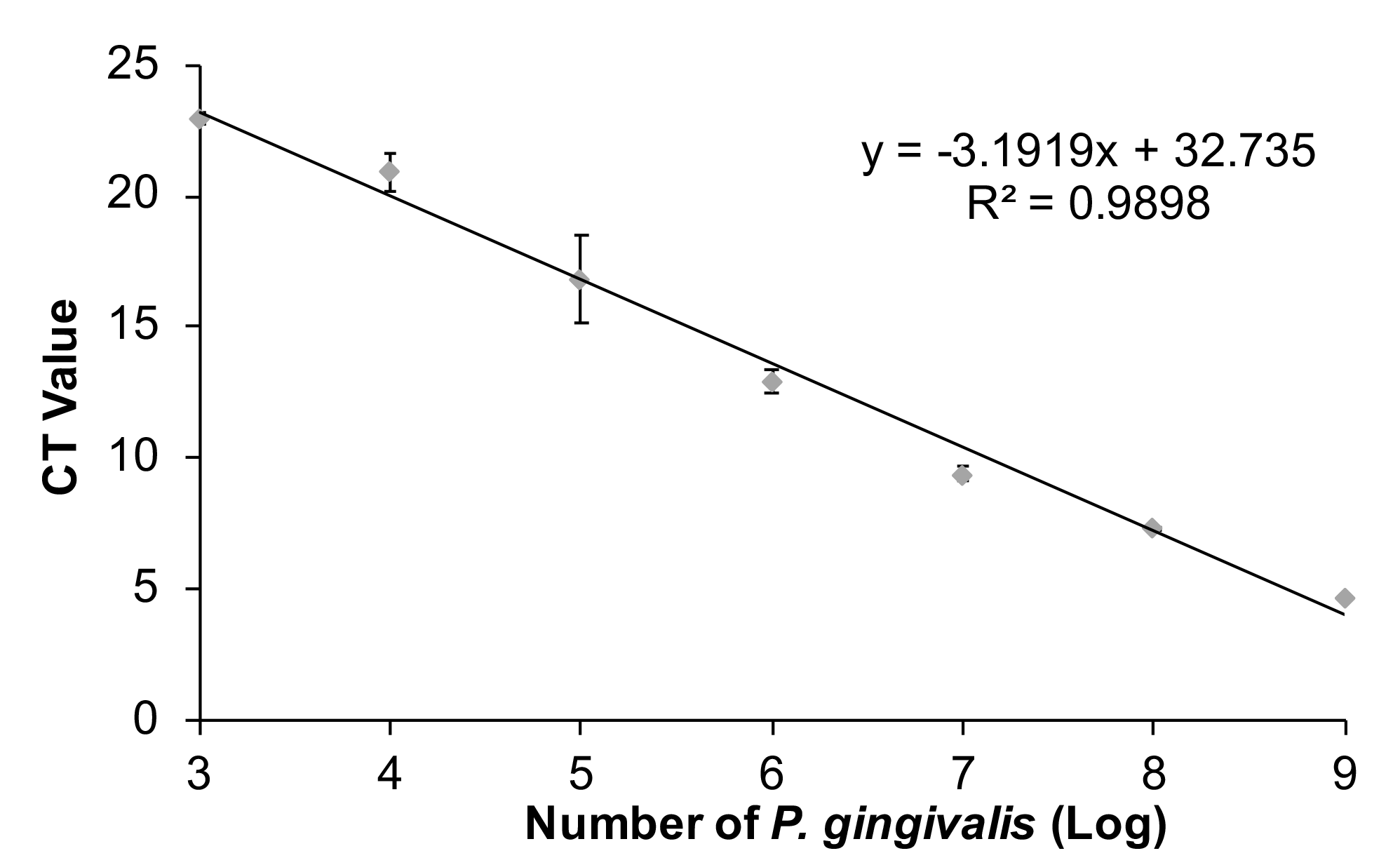

Supplement: Supplementary Figure 2 — qPCR standard curve of P. gingivalis. [file Image_2.tif]

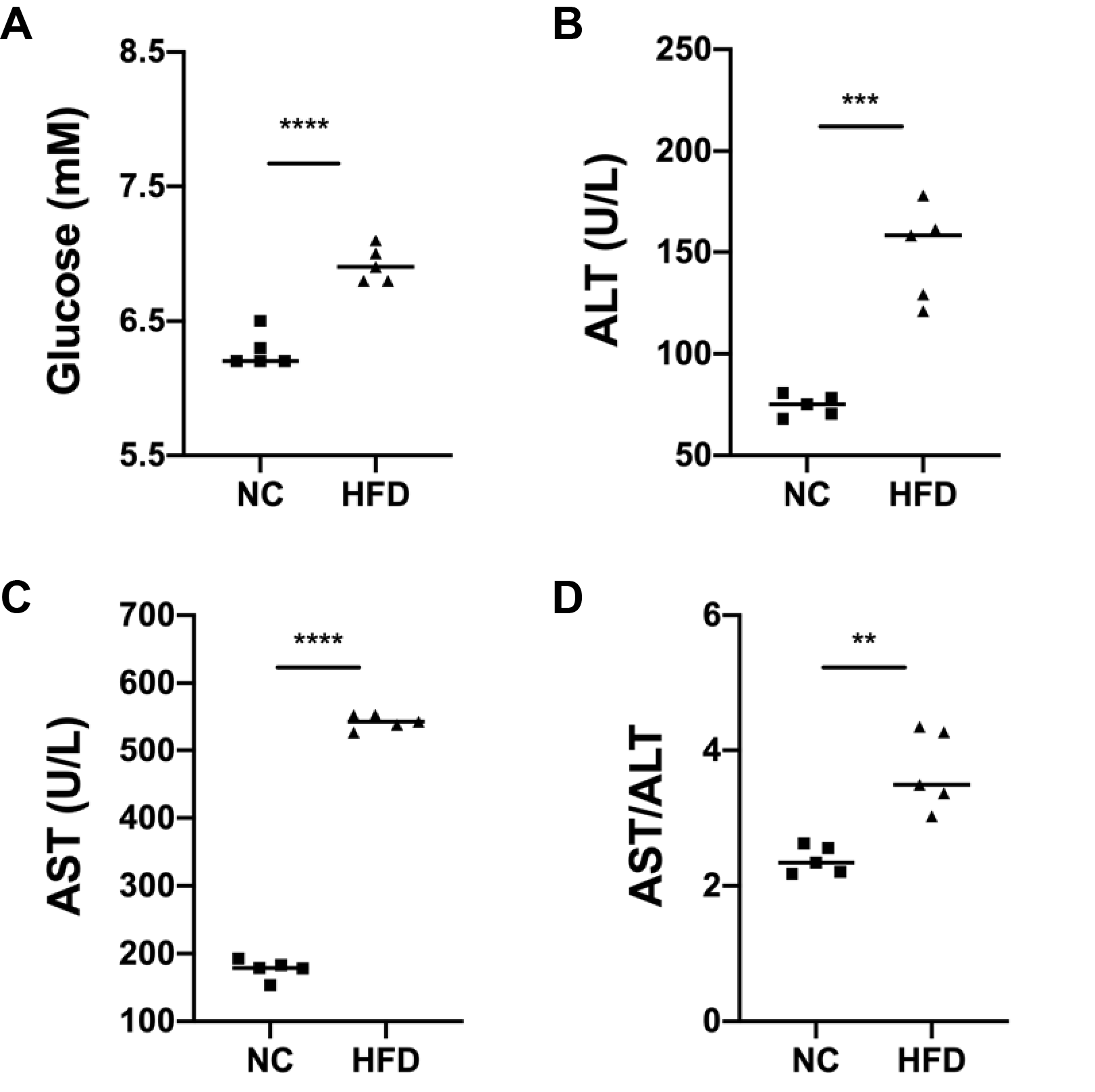

Supplement: Supplementary Figure 3 — Effect of HFD on liver function. Serum (A) glucose, (B) ALT, (C) AST levels, and (D) AST/ALT ratio in HFD-fed mice were compared with those in NC-fed mice. HFD, high-fat diet; NC, normal chow. **P ≤ 0.01; ***P ≤ 0.001; ****P ≤ 0.0001. [file Image_3.tif]
