# Supplementary material for: Porphyromonas gingivalis Induces Increases in Branched-Chain Amino Acid Levels and Exacerbates Liver Injury Through livh/livk
Source: Front Cell Infect Microbiol. 2022 Mar 10;12:776996. doi: 10.3389/fcimb.2022.776996 (PMC8961321; doi:10.3389/fcimb.2022.776996)
Supplement: Supplementary file 4 [file Table_1.docx]

|  | **Table S1. Primers used in this study** |  |
| --- | --- | --- |
| Name | Primer sequence (5’ – 3’) | Function |
| RTlivhF | ATGGTTTATACGGCGAGCGT | Reverse-transcriptase PCR primers to determine *livh* expression |
| RTlivhR | ATCTGCCAACTCTCAAGCCC |  |
| RTlivkF | AGTGATTGGAGGCAATGGCA | Reverse-transcriptase PCR primers to determine *livk* expression |
| RTlivkR | GCTGATTAAAAGCACCGCCT |  |
| 16S rRNAF | TACCCATCGTCGCCTTGGT | Reverse-transcriptase PCR primers to determine *16S rRNA* expression |
| 16S rRNAR | CGGACTAAAACCGCATACACTTG |  |
| livh-UF | CTGGTGACCATGTGTATATAAT | Generation of upstream of livh |
| livh-UR | ACGAACGGGCAATTTCTTTTTTGTCATCTTATTTGGGGCTTTTATTAGG |  |
| livh-DF | GATGGAGCGGAAACGTAAAAGATGGAGTATTTGCTACATCTG | Generation of downstream of livh |
| livh-DR | AAGGCTTCGGGAAGTAAG |  |
| livk-UF | ATAACATCAAACAATTCATCGT | Generation of upstream of livk |
| livk-UR | ACGAACGGGCAATTTCTTTTTTGTCATTCTCTAAAAGCTTTATAGC |  |
| livk-DF | GATGGAGCGGAAACGTAAAAGATCACACAACAAGAAGTTATTG | Generation of downstream of livk |
| livk-DR | TATCAAAGACTCGATTGTAG |  |
| Erm-F | ATGACAAAAAAGAAATTGCCCGT | Generation of Erm |
| Erm-R | TCTTTTACGTTTCCGCTCCATC |  |
|  |  |  |
