# Supplementary material for: Porphyromonas gingivalis Induces Increases in Branched-Chain Amino Acid Levels and Exacerbates Liver Injury Through livh/livk
Source: Front Cell Infect Microbiol. 2022 Mar 10;12:776996. doi: 10.3389/fcimb.2022.776996 (PMC8961321; doi:10.3389/fcimb.2022.776996)
Supplement: Supplementary Table 2 — The compositions of D12492 and D12450J. [file Table_2.docx]

**Table S2 Feed formula of D12492 and D12450J**

|  | D12492 | | D12450J | |
| --- | --- | --- | --- | --- |
| Ingredient | g | kcal | g | kcal |
| Casein, 80Mesh | 200 | 800 | 0 | 0 |
| Casein, 30Mesh | 0 | 0 | 200 | 800 |
| L-Cystine | 3 | 12 | 3 | 12 |
| Corn Starch | 0 | 0 | 506.2 | 2024.8 |
| Maltodextrin 10 | 125 | 500 | 125 | 500 |
| Sucrose | 68.8 | 275.2 | 68.8 | 275.2 |
| Cellulose, BW200 | 50 | 0 | 50 | 0 |
| Soybean Oil | 25 | 225 | 25 | 225 |
| Lard | 245 | 2205 | 20 | 180 |
| Mineral Mix, S10026 | 10 | 0 | 10 | 0 |
| DiCalclum Phosphate | 13 | 0 | 13 | 0 |
| Calcium Carbonate | 5.5 | 0 | 5.5 | 0 |
| Potassium Citrate, 1 H20 | 16.5 | 0 | 16.5 | 0 |
| Vitamin Mix, V10001 | 10 | 40 | 10 | 40 |
| Choline Bitartrate | 2 | 0 | 2 | 0 |
| FD&C Blue Dye #1 | 0.05 | 0 | 0.01 | 0 |
| FD&C Yellow Dye #5 | 0 | 0 | 0.04 | 0 |
| Total | 773.85 | 4057 | 1055.05 | 4057 |
