# Supplementary material for: Porphyromonas gingivalis Induces Increases in Branched-Chain Amino Acid Levels and Exacerbates Liver Injury Through livh/livk
Source: Front Cell Infect Microbiol. 2022 Mar 10;12:776996. doi: 10.3389/fcimb.2022.776996 (PMC8961321; doi:10.3389/fcimb.2022.776996)
Supplement: Supplementary file 8 [file Table_5.docx]

**Table S5 Body weight of the mice at the baseline and the end of experiment**

|  | Baseline (mean±SD g) | End of experiment (mean±SD g) |
| --- | --- | --- |
| NC | 19.20±1.05 | 28.62±1.18 |
| HF | 19.16±0.93 | 35.20±3.63^***^ |
| WT+HFD | 19.72±0.89 | 36.87±2.75 |
| *△livh* +HFD | 19.09±1.02 | 35.86±2.91 |
| *△livk* +HFD | 19.31±0.81 | 34.75±2.13 |
